# Supplementary material for: Ionic Liquids Enhanced Alkynyl Schiff Bases Derivatives of Fipronil Synthesis and Their Cytotoxicity Studies
Source: Molecules. 2019 Sep 4;24(18):3223. doi: 10.3390/molecules24183223 (PMC6767227; doi:10.3390/molecules24183223)
Supplement: Supplementary file 1 [file molecules-24-03223-s001.pdf]

Ionic Liquids Enhanced Alkynyl Schiff Bases Derivatives of Fipronil Synthesis and Their Cytotoxicity Studies

Xiu Liu[1,2,*], Linya Huang[1], Hongjun Chen[3], Na Li[2], Chao Yan[2], Chenzhong Jin[1], Hanhong Xu[2*]

1 Key Laboratory of Pesticide Harmless Application, Collaborative Innovation Center for Field Weeds Control (CICFWC) of Hunan Province, Hunan University of Humanities, Science and Technology, Loudi, 417000, P. R. China; 1436431145@qq.com (L.H.); 532479626@qq.com (C.J.);

2 Key Laboratory of Natural Pesticide and Chemical Biology, Ministry of Education, South China Agricultural University, Guangzhou 510642, P. R. China; 25269893@qq.com (N.L.); 79645713@qq.com (C.Y.);

3 Hunan Provincial Key Laboratory of Fine Ceramics and Powder Materials, School of Materials and Environmental Engineering, Hunan University of Humanities, Science and Technology, Loudi, 417000, P. R. China; hongjunchen@hnu.edu.cn (H.C.);

* Correspondence: liuxiu841027@163.com (X.L.); hhxu@scau.edu.cn (H.X.); Tel.: +86-20-8528-5127 (H.X.)

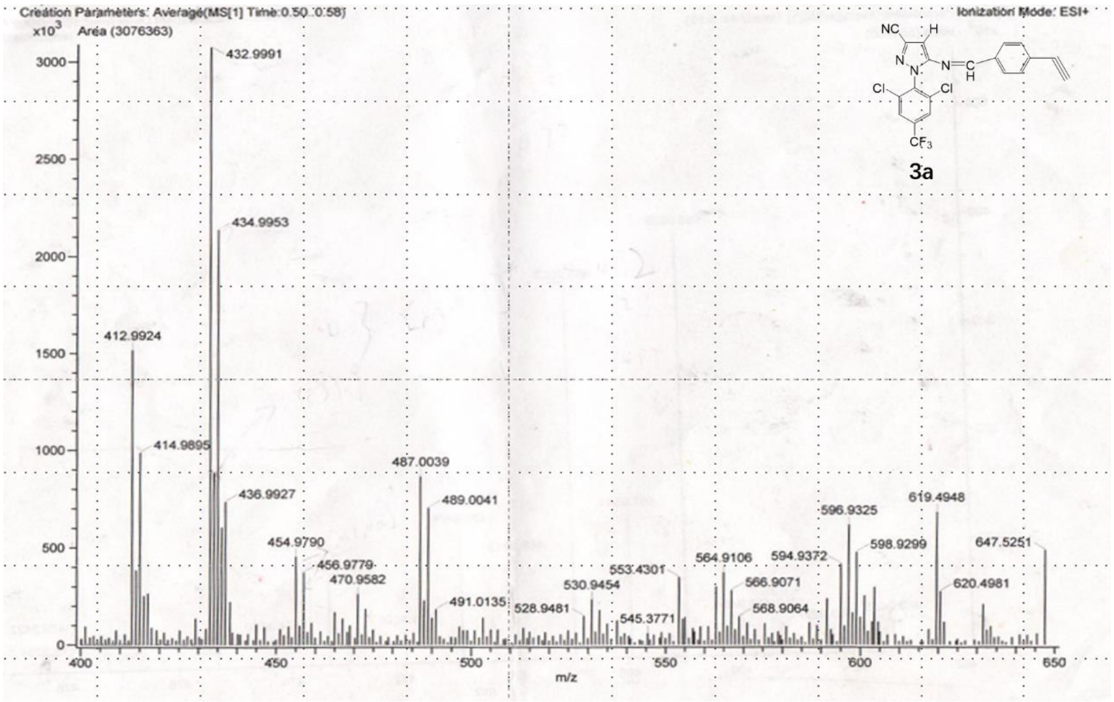

Figure S1. ESI-HRMS spectrum of 1-(2,6-dichloro-4-(trifluoromethyl)phenyl)-5-(4-ethynylbenzylideneamino)-1H-pyrazole-3-carbonitrile (**3a**).

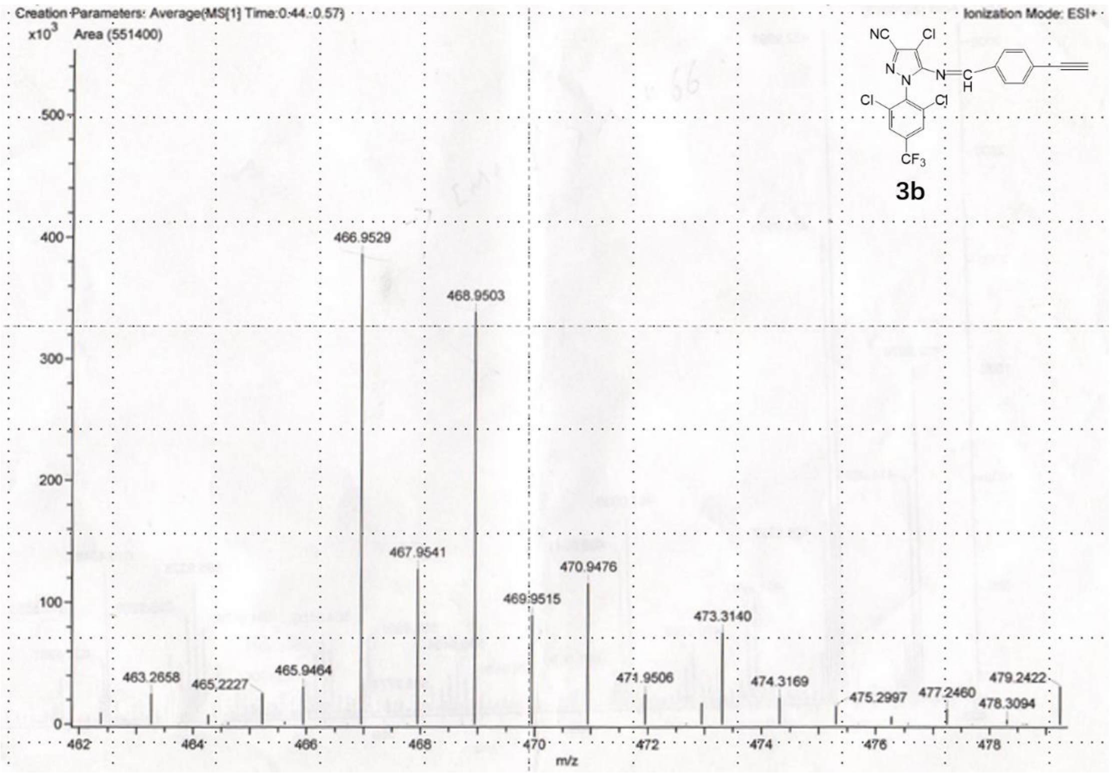

Figure S2. ESI-HRMS spectrum of 4-chloro-1-(2,6-dichloro-4-(trifluoromethyl)phenyl)-5-(4-ethynylbenzylidene-amino)-1H-pyrazole -3-carbonitrile (**3b**).

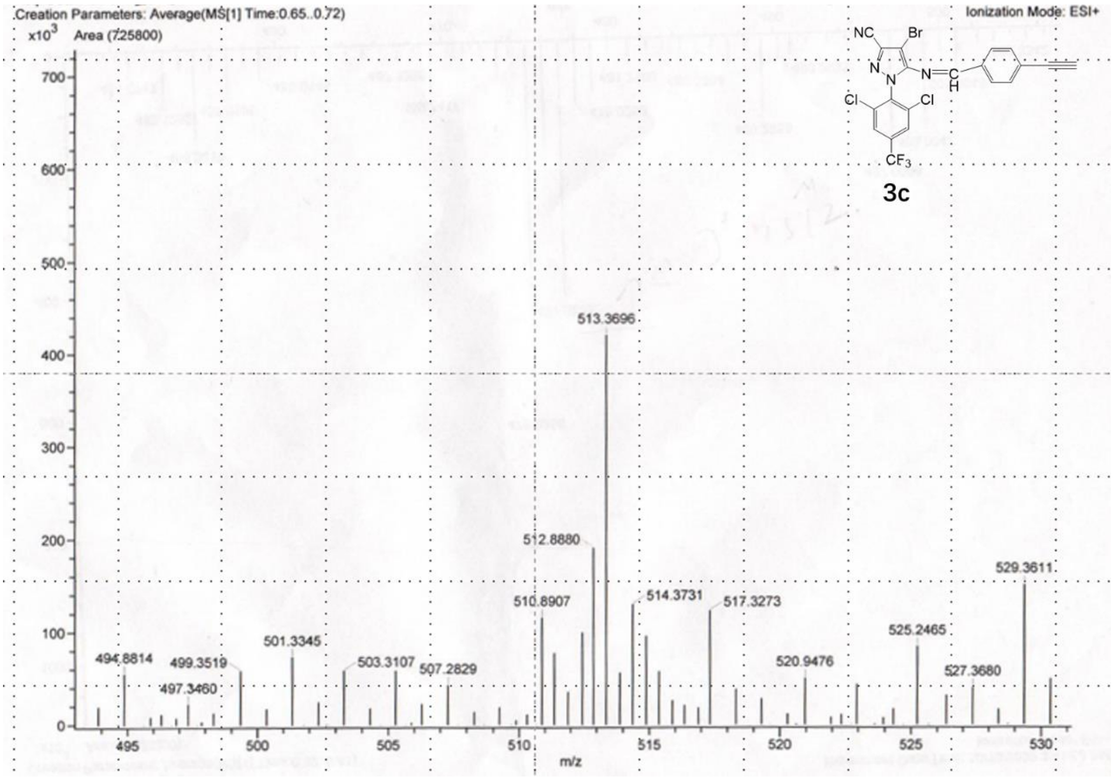

Figure S3. ESI-HRMS spectrum of 4-bromo-1-(2,6-dichloro-4-(trifluoromethyl)phenyl)-5-(4-ethynylbenzylidene-amino)-1H-pyrazole -3-carbonitrile (**3c**).

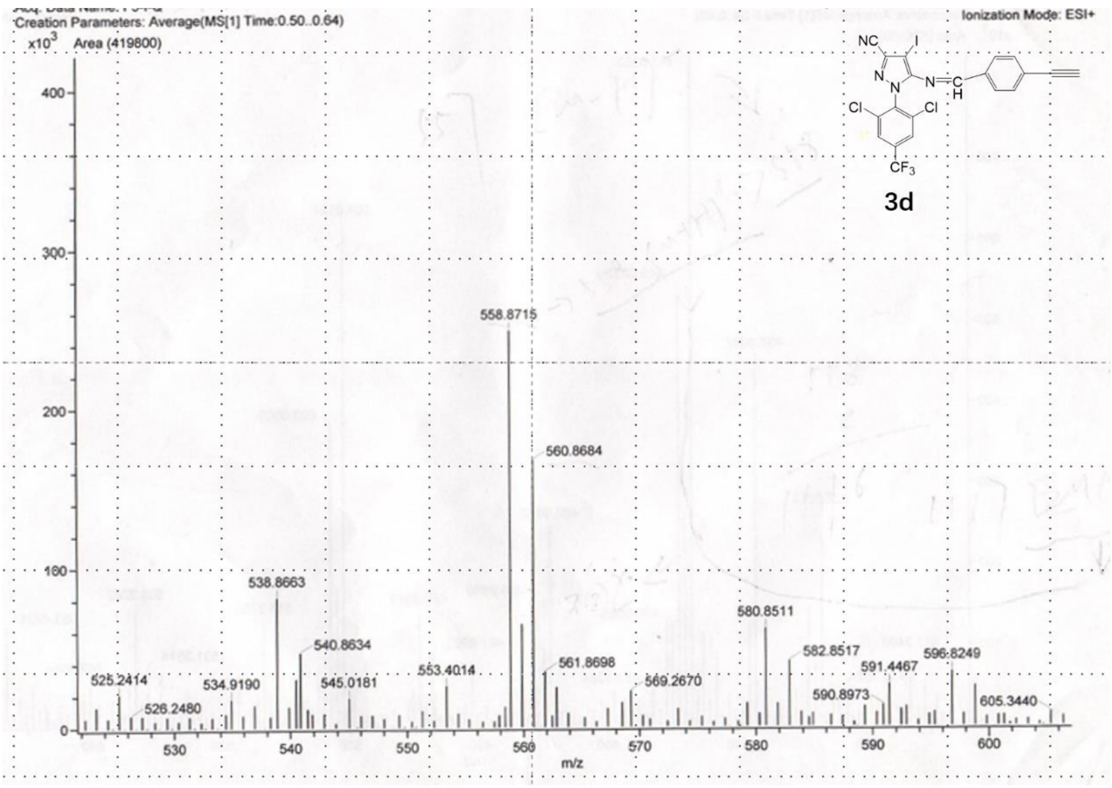

Figure S4. ESI-HRMS spectrum of 4-iodo-1-(2,6-dichloro-4-(trifluoromethyl)phenyl)-5-(4-ethynylbenzylidene-amino)-1H-pyrazole -3-carbonitrile (**3d**).

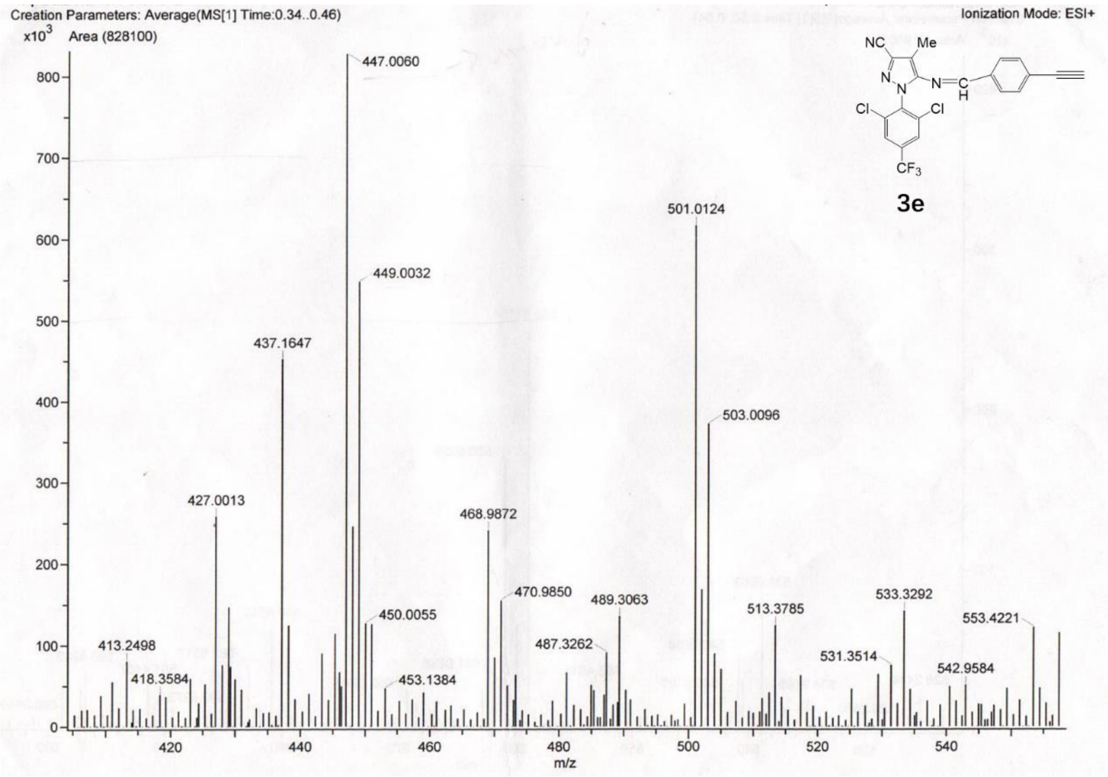

Figure S5. ESI-HRMS spectrum of 1-(2,6-dichloro-4-(trifluoromethyl)phenyl)-5-(4-ethynylbenzylideneamino)-4-methyl-1H-pyrazole -3-carbonitrile (**3e**).

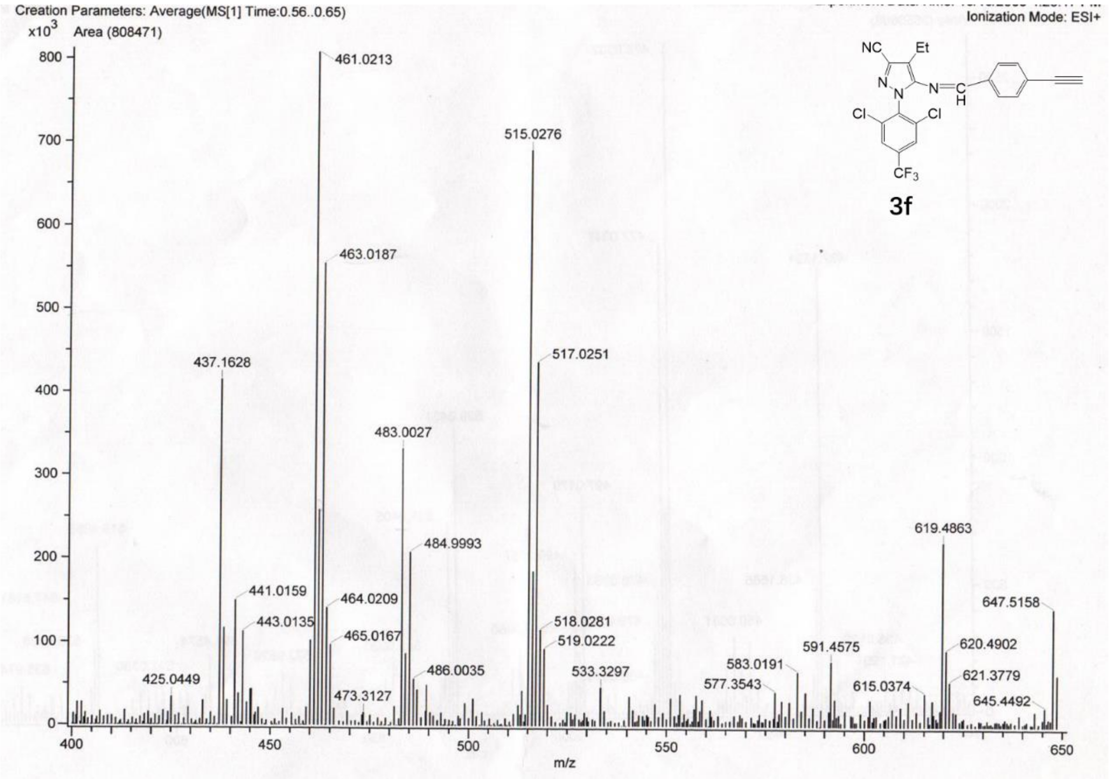

Figure S6. ESI-HRMS spectrum of 1-(2,6-dichloro-4-(trifluoromethyl)phenyl)-5-(4-ethynylbenzylideneamino)-4-ethyl-1H-pyrazole -3-carbonitrile (**3f**).

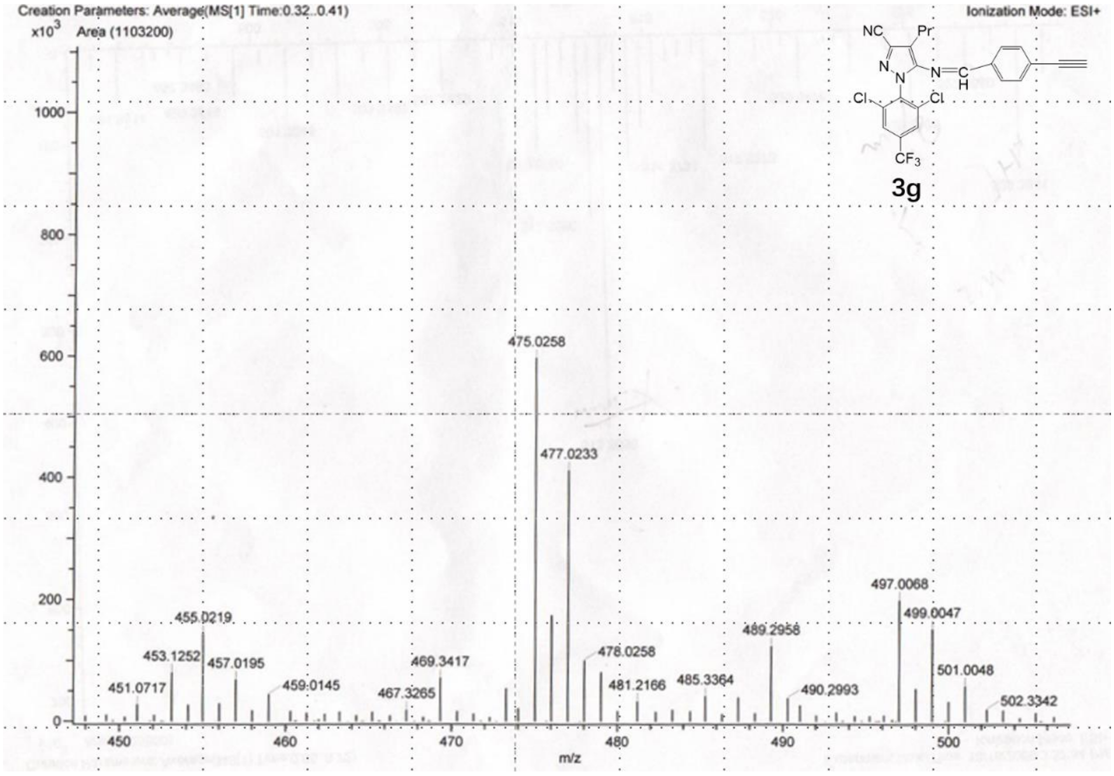

Figure S7. ESI-HRMS spectrum of 1-(2,6-dichloro-4-(trifluoromethyl)phenyl)-5-(4-ethynylbenzylideneamino)-4-propyl-1H-pyrazole -3-carbonitrile (**3g**).

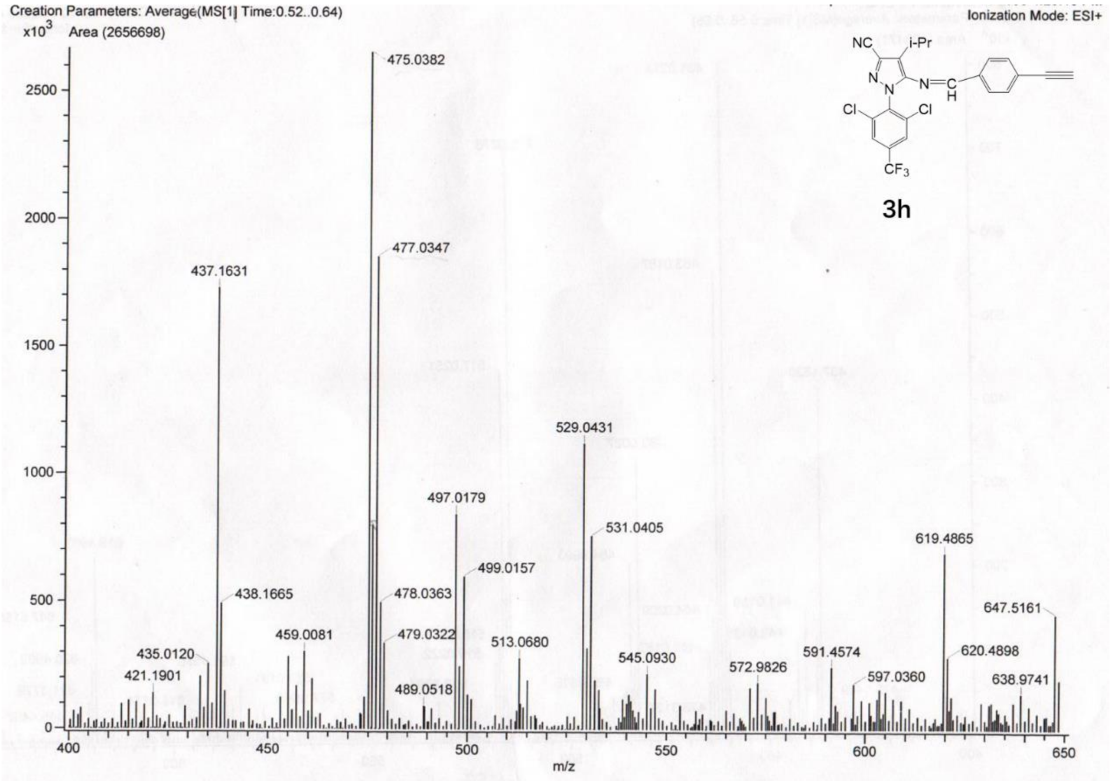

Figure S8. ESI-HRMS spectrum of 1-(2,6-dichloro-4-(trifluoromethyl)phenyl)-5-(4-ethynylbenzylideneamino)-4-isopropyl-1H-pyrazole -3-carbonitrile (**3h**).

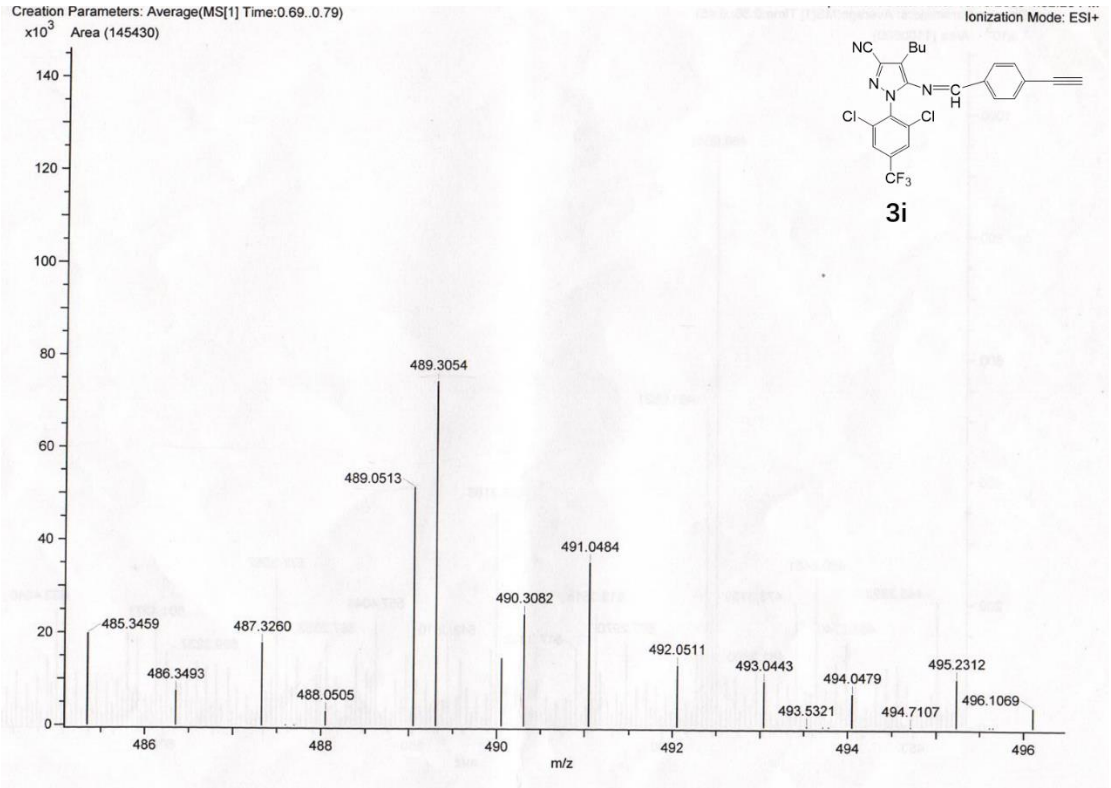

Figure S9. ESI-HRMS spectrum of 1-(2,6-dichloro-4-(trifluoromethyl)phenyl)-5-(4-ethynylbenzylideneamino)-4-butyl-1H-pyrazole -3-carbonitrile (**3i**).

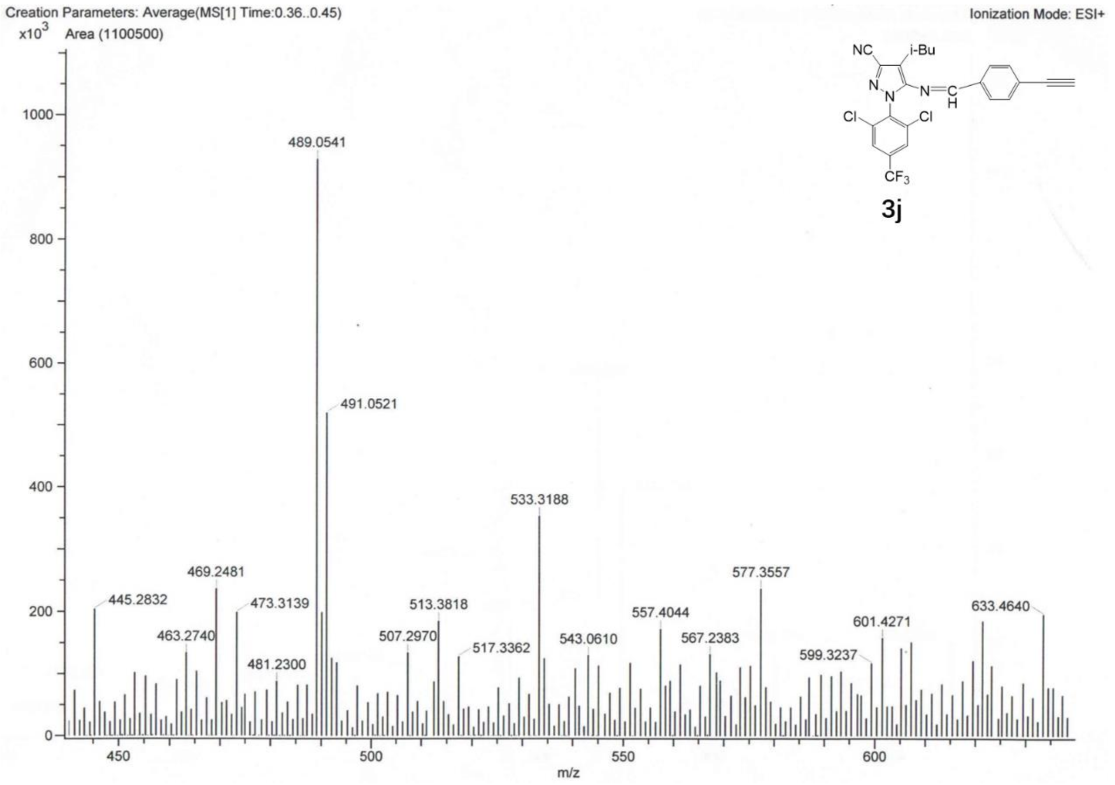

Figure S10. ESI-HRMS spectrum of 1-(2,6-dichloro-4-(trifluoromethyl)phenyl)-5-(4-ethynylbenzylideneamino)-4-isobutyl-1H-pyrazole -3-carbonitrile (**3j**).

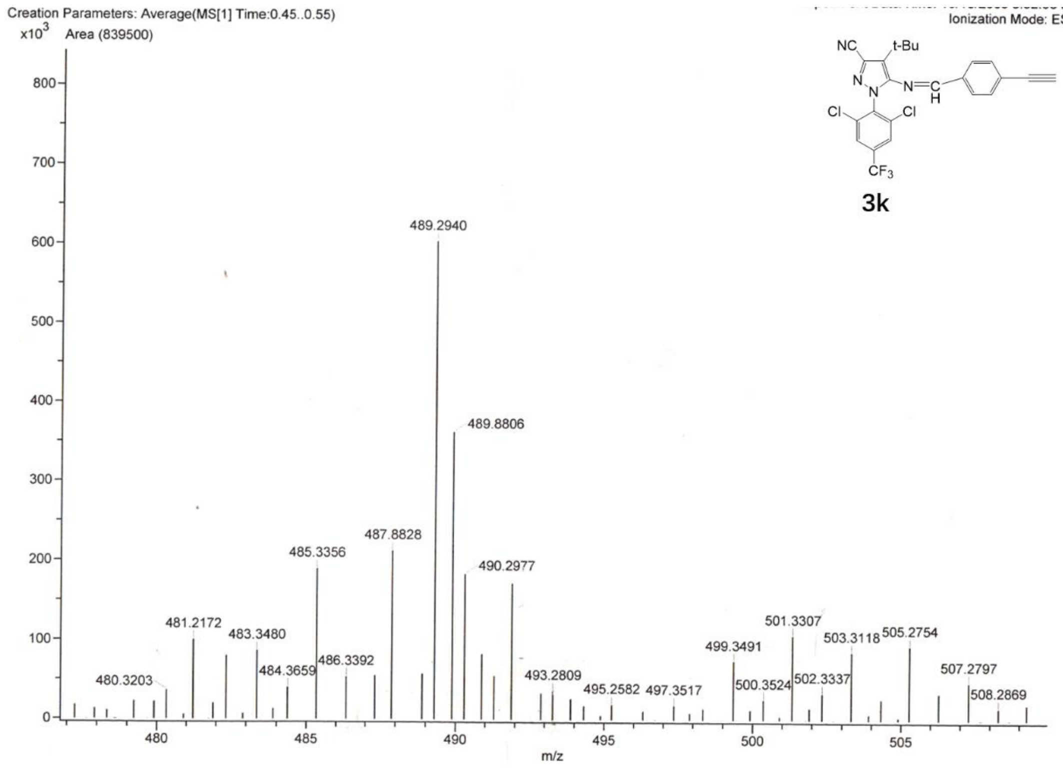

Figure S11. ESI-HRMS spectrum of 1-(2,6-dichloro-4-(trifluoromethyl)phenyl)-5-(4-ethynylbenzylideneamino)-4-tert-butyl-1H-pyrazole -3-carbonitrile (**3k**).
